# The social context of adolescent women’s use of modern contraceptives in Zimbabwe: a multilevel analysis

**DOI:** 10.1186/1742-4755-11-64

**Published:** 2014-08-10

**Authors:** Enock Ngome, Clifford Odimegwu

**Affiliations:** 1 Department of Population Studies, University of Botswana, Private Bag UB0022, Gaborone, Botswana; 2 Demography and Population Studies Programme, University of the Witwatersrand, Private Bag 3, WITS 2050 Witwatersrand, South Africa

**Keywords:** Contraceptive use, Adolescent women, Socioeconomic development, Multilevel analysis, Zimbabwe

## Abstract

**Background:**

Efforts aimed at reducing maternal mortality as per the Millennium Development Goal 5 (MDG 5) include reducing early childbearing through increased adolescent contraceptive use. Despite a substantial attempt to study factors influencing adolescent contraceptive use in Sub-Saharan Africa (SSA), few studies have explored the role of community level characteristics on adolescent modern contraceptive use. This study examines the influence of both individual, household and community variables in influencing adolescent contraceptive use in Zimbabwe. This study posits that community characteristics are more critical predictors of adolescent contraceptive use in Zimbabwe than other individual and household characteristics.

**Methods:**

Data from the 2010/11 Zimbabwe Demographic Health Survey (ZDHS), supplemented by additional data from the Measure DHS consultants were used. A total weighted sample of 457 non-pregnant adolescent women aged 15 to 19 years who had their last sex within 12 months preceding the 2010/11 ZDHS was analysed. Univariate, bivariate and multilevel binary logistic regression analysis were performed using generalized linear mixed models (GLMM).

**Results:**

The odds of contraceptive use were higher for adolescent women with one or more children ever born (Odds Ratio (OR), 13.6) and for those ever married (OR, 2.5). Having medium and high access to media also increased the odds of using contraceptives (OR, 1.8; 2.1 respectively). At community level, the odds of modern contraceptive use decreased with an increase in the mean number of children ever borne per woman (OR, 0.071), an increase in the mean number of school years per women (OR, 0.4) and an increase in the proportion of women with at least secondary education (OR, 0.5). It however increased with an increase in the proportion of women experiencing at least one problem accessing health care (OR, 2.0). Individual and community level variables considered successfully explained the variation of adolescent contraceptive use across provinces.

**Conclusions:**

Both individual and community characteristics were important predictors of adolescent contraceptive use in Zimbabwe. Reproductive program interventions aimed at increasing adolescent contraceptive use should take into account both individual and community factors. There is need for further research that examines other community characteristics influences that include political and cultural factors.

## Introduction

The high rates of early childbearing among women in Sub-Saharan African (SSA) countries continue to be a public health concern. It is estimated that about 16 million of women under 20 years give birth each year and this constitutes 11 percent of all births globally. The developing countries contribute 95% of all births by adolescents [1]. Early childbearing impacts negatively on adolescent women through impairment of their health and that of their offspring [2]. It is also associated with increased risk of adverse pregnancy outcomes and infant mortality [3-5]. In developing countries, complications during pregnancy and childbirth have been identified as the leading cause of death among adolescent girls [6]. In addition, studies have shown that adolescent women have a high risk of experiencing an unintended pregnancy. Unplanned pregnancies have been associated with negative health risk factors for both the mother and the child [2,7,8]. The use of modern contraceptives has thus been identified as one of the main interventions to reduce the negative effects of early childbearing. This study sought to investigate the social context of adolescent women’s use of modern contraceptives on Zimbabwe.

Zimbabwe has one of the most successful family planning programs in sub-Saharan Africa (SSA). Together with Rwanda, the have the lowest unmet need for contraception among adolescent women in Africa. Despite this, there has been concern of an increasing unmet need for contraception among adolescent during the last decade and a half. Unmet need for contraception among the married adolescent women increased from 13% during the 1999 Zimbabwe Demographic Health Survey (ZDHS) to 18.5% during the 2010/11 ZDHS whereas it increased from 41% to 53% among the unmarried during the same period [9]. Therefore, the need to understand factors influencing contraceptive use among adolescent women becomes crucial to inform reproductive health intervention in Zimbabwe.

Extensive research has been made to understand individual level factors influencing the uptake of modern contraceptives. In particular, socioeconomic and demographic factors such as residence [10], education [11-13], age [11,14], economic status [15], parity [16], access to media [13,17], autonomy [18-20], desire for children [20], marital status [10] have been associated with use modern contraceptives. Partner communication has also been identified as an important factor influencing contraceptive use [16,21]. Women whose partners disapprove of modern contraceptive practice are more likely not to use modern contraceptives [13,20]. Psychosocial factors such as intimate partner violence have also been associated with non-use of contraception among women [22]. These studies however neglected the importance of the influence of community based characteristics on use of modern contraceptives.

Studies on the importance of examining community level factors in influencing contraceptive use are still limited [14,23-25]. Such studies have focused on the influence of health service characteristics and in particular, the quality of care on contraceptive adoption [26-30]. Other characteristics of the community that have been associated with contraceptive use include community economic development [31-35], school participation levels [36,37], children’s economic roles [38,39] and community fertility norms [31,33,40-42]. Some studies however demonstrate the importance of considering diffusion effects on many expected individual socioeconomic factors that influence contraceptive use at levels beyond women’s own individual circumstances [43].

Mostly, studies on the influence of community characteristics are in developed countries. In addition, existing studies ignored that the individual effect on contraceptive use might be compromised by some aspects of the community that may not be understood. These studies fail to establish the independence of the community variables from the individual variables. Such independence has been established by other studies [34,44] which explains the significantly huge cluster variation that remains even after controlling for the effect of the individual characteristics. This study builds on previous studies by advancing existing knowledge beyond understanding of individual and household determinants of contraceptive use. The premise of this study is that a combination of socioeconomic development, quality of reproductive health and access to health care services of the community in which an adolescent woman lives tend to modify individual level behavioral factors. Building on this, we explore the levels and influences of contextual determinants of contraceptive use among the adolescents in Zimbabwe using multilevel approach. Multilevel analysis allows simultaneous examination of community level and individual level characteristics [45]. The community characteristics that are considered in the study include the influence of quality of health care, access to health care and socioeconomic development of the communities in which adolescent women live.

## Methods

### Study area and data source

Zimbabwe is the study area. Administratively, Zimbabwe is divided into eight provinces and 2 cities with provincial status and these are Harare, Bulawayo, Matebeleland North, Matebeleland South, Manicaland, Mashonaland Central, Mashonaland East, Mashonaland West, Midlands and Masvingo. The data source is a nationally representative 2010–11 Zimbabwe Demographic Health Survey (ZDHS). The survey was designed to provide population and health indicators estimates at national and provincial levels. The 2010/11 ZDHS data was supplemented by additional data from the Measure DHS consultants to assign provincial values to cases for multilevel modelling (see Statistical Analysis for multilevel modelling).

Women aged 15 to 49 years who were found in selected households were individually interviewed on issues of fertility, family planning and maternal and child health. A total of 9171 women were interviewed of which 21.2% (1945) were adolescent women aged 15 to 19 years. For this study, only a sample of adolescent women who had their last sex within 12 months prior to the survey was used for the analysis. Excluded from the sample included those who reported being pregnant at the time of the ZDHS survey, who did not know the timing of their last sexual activity and those who had their last sex beyond 12 months preceding the survey. Out of a weighted sample of 1945 adolescent women, 23.5% (457) reported to have had their last sex within 12 months prior to the survey and did not report to be pregnant at the time of the survey.

### Dependent (outcome) variable

The dependent variable for this study is whether adolescents (15 to 19 years) are current users of modern contraceptives. During the survey, sexually active women were asked if they were currently doing something or using any method to delay or avoid getting pregnant. Those that report doing something or using any method to delay or avoid getting pregnant were further asked to indicate what they are doing or the method they are using. Women were then categorized as those using modern contraceptive methods (using pill, IUD, Injection, Implants, Male/Female Condom, Diaphragm or Lactational Amenorrhea Method) and those that don’t use modern contraceptive methods (either using withdrawal, periodic abstinence, folklore method or nothing at all). Those using modern contraceptives were coded as 1, otherwise they were coded as 0.

### Independent variables

Individual-level variables for this study included woman’s autonomy, household wealth index, age, marital status, age difference between woman and spouse, parity, level of education, access to media and residence. To measure women’s autonomy, a composite variable depicting the level of women’s autonomy and decision making was derived from a selected four dimensions of women’s autonomy derived from questions from the 2010/11 ZDHS: Their participation in household decision making on health care of self, large household purchases, daily household purchases, and visiting families or relatives. Responses to these questions are coded 1) Respondent, 2) Respondent and Husband/Partner Jointly, 3) Respondent and Someone else, 4) Husband/Partner and 5) Someone Else. A dummy variable was created such that any respondent who indicated being involved in the decision making is coded as 1 otherwise its coded 0 (whether the decision is jointly or by the husband/partner alone). This was done by collapsing the first three categories (response 1, 2 and 3) and given score 1 since the respondent was involved in the decision making process, otherwise 0 score was awarded to the other categories. Summing up all the scores for each dimension of women’s autonomy provided the level scales of women’s autonomy ranging from 0 to 4. Women with scale 4 were considered to be highly autonomous and those with 3 or less were considered to have low autonomy as they reported not participating in at least one aspect of household decision making. This is good measure of the women’s autonomy since health care utilization can be hampered one way or the other by the lack of independence a woman has in any of the aspects considered.

The household wealth index was derived through Principal Component Analysis from 10 household possessions (radio, television, mobile telephone, non-mobile telephone, refrigerator, bicycle, motorcycle/motor scooter, animal drawn cart, a car/truck and a boat with a motor) and household assets/amenities (drinking water, toilet facility, availability of electricity, floor material, fuel for cooking and ownership of land). To determine the index, each of the items was assigned a factor score and then individual were ranked according to the total factor score of the household they live. This information is then used to come up with a household asset index using the Principal Component Analysis (PCA) [46]. The household wealth index is in quintiles and is used to estimate a household’s economic wellbeing. These were further categorized into low wealth (Poor and Poorest quintiles), medium wealth (Medium quintile) and high wealth (Rich and Richest quintiles).

The age is the self-reported age of the respondent at the time of the survey and was categorized into 15 to 17 years and 18 to 19 years. Marital status was also categorized into two (Never Married and Ever Married (the ever married combined the married, divorced, separated and widowed). The age difference between woman and spouse refers to the absolute difference between the age of the husband/partner and that of the adolescent woman. This was created by subtracting the age of the adolescent woman at the time of the survey from that of the husband/partner at the time of the survey. Age difference between woman and spouse was categorised into large difference and small or no difference. Large difference refers to the age difference which is five years and above and small or no difference refers to the age difference which is less than five years.

Parity is the number of live births that a woman has ever had. This indicator was categorized into two, being none (those with no children ever born (CEB)) and one or more CEB. The level of education denotes the highest level of education attained by the adolescent women at the time of the survey. Due to the small number of adolescent women who have never been to formal education, the education level of woman has been categorized into two. Those with no formal education were put together with those with primary school as their highest level of education. The other category represented all women with secondary school level as the highest level of education and above. Religion represents the self-reported religious group at the time of the survey. This has been categorized into the following 3 groups: Those affiliated to the Protestant and Pentecostal were categorized together and coded as 0, those belonging to the Traditional and Apostolic Sect put together and coded 1 and the last group is Other Religions and was coded 2. Other groups include the Catholics, Muslim, Other Christians and those not affiliated to any religion.

Media access is a composite variable derived from the following 3 questions asked to the respondents; 1) Do you read a newspaper or magazine almost every day, at least once a week, less than once a week or not at all?, 2) Do you listen to the radio almost every day, at least once a week, less than once a week or not at all?, 3) Do you watch television almost every day, at least once a week, less than once a week or not at all?. Responses to all the three questions were coded as follows: 0) Not at all, 1) Less than once a week, 2) At least once a Week, 3) Almost Every day. To develop the composite variable, a Cronbach’s Alpha was estimated since it is a good measure of internal consistency. The Cronbach’s Alpha was 0.749 which implies that about 75% of the variability in a composite score by combining the 3 variables used to measure access to media, will be considered as reliable score variance. Any Cronbach’s Alpha estimate of 0.7 and above is considered a reliable measure of internal consistency [47,48]. The codes for each of the three questions were used as scores and all the scores from the three questions were summed up for each respondent to create a level of access to media score. The score ranged from 0 to 9 was then collapsed into three categories indicating the level of access to media. Respondents with scores ranging from 4 to 9 were regarded as having high access to media; those with scores ranging from 1 to 3 were regarded as having medium access to media. Those who scored 0 were regarded as having no access to media. Residence was categorized according to whether the respondent resides in a rural or an urban area.

Community level variables were measured at provincial level as most policy decisions influencing reproductive health care issues are taken at this level. Variables included quality to reproductive health care index, access to health care and socio-economic development indicators. The 2010/11 ZDHS data source was used to derive community (provincial) level variables and then provincial values were assigned to each adolescent woman belonging to their respective provinces respectively for multilevel modelling (see statistical analysis). To measure the quality of reproductive health care, a composite variable depicting quality to reproductive health care at community (Provincial) level was created to come up with an average number of reproductive health care services given to women. Reproductive health care service questions used include examinations done during antenatal care (ANC), the counseling or health education given to females, treatment and supplies given, and type of assistance during delivery and postnatal care (PNC).

During the survey, respondents were asked whether the following had been done at least once on them; blood pressure measured, urine sample taken and blood sample taken as part of the examination during the ANC. The three variables were coded “Yes” versus “No”. On counselling or health education, respondents were also asked whether they were told about the signs of pregnancy complications. The variable was coded “Yes” against “No/Don’t Know”. With regard to treatment and supplies, respondents were asked if they were given any injection in the arm to prevent the baby from getting tetanus, that is convulsions after birth, whether they given or bought any iron tablets during the pregnancy, and whether they were given or bought intestinal parasite drugs during pregnancy. Coding for the two variables were “Yes” versus “No/Don’t Know”. These activities are in line with the WHO recommendations [49] to improve maternal health care among women.

The last two variables involved in measuring quality to health care entailed assistance from qualified or skilled health personnel during delivery of last child five years preceding the survey, and a health check by skilled health care personnel before discharge after delivery of last child. Coding for this variable was “Skilled Health Personnel (doctor, nurse/midwife) versus other unskilled personnel (traditional birth attendant, relative friend, no assistance or any other person who is not health personnel). The variable was coded “Yes” against “No”.

For each of the nine services provided, a score of 1 was awarded otherwise a score of 0 was given. A score depicting the number of reproductive health services provided for each woman was derived by summing up the scores awarded for each service provided and this ranged from 0 to 9. The quality of health care index was created by averaging the number of reproductive health care services provided for women in each community (Province). The closer the index the index is to 9 the higher the quality of reproductive health services in the Province.

Access to health care was measured through by using women’s experience in four areas that included getting permission to go for treatment, getting money for treatment, distance to a health facility, and having to take transportation. These questions ensure that barriers to access cover a wide range of areas such as ability to go for treatment, affordability, distance to health facility and mobility (transportation). From these questions, the proportion of women experiencing at least one of the problems accessing health care was derived. This was done for each community (Province). The higher the proportion of women experiencing at least one problem accessing health care, the lower the level of access to health care for women in the Province.

The following variables were used to indicate the socioeconomic development at community level; mean number of children ever borne per woman, mean number of years of school for women, proportion of females with at least secondary education, proportion of women with partners approving of use of contraception, contraceptive prevalence rate (CPR), proportion of women accessing antenatal care (ANC) at least once, proportion of women who delivered their last child in a health facility and provincial proportion of women participating in labour force. Table 1 shows individual and household level variables, and community level variables and their operational definitions.

**Table 1:** Individual, household and community variables used in modeling adolescent modern contraceptive use in Zimbabwe

	**Operational definition and coding**
***Individual and household variables***	
• Age	• Self-reported age of respondent at the time of the survey [15 to 17 Years/18 to 19 Years]
• Marital status	• Marital status of respondent [(Never Married; Ever Married (married/separated/divorced/widowed)]
• Age difference with partner/spouse	• The absolute difference between the self-reported age of respondent and the given age of the husband/spouse/partner [Large; Small or None]
• Parity	• Number of Children ever given birth [None; One or more]
• Education	• Highest Level of Education attained [None/Primary; Secondary or Higher]
• Religious affiliation	• Self-reported Religious Group [Protestant/Pentecostal, Traditional/Apostolic Sect, Catholics/Muslim/Other/None]
• Residence	• Current Place of Residence [Urban; Rural]
• Media access	• A composite variable derived from the frequency of access to newspaper/magazine, radio and television [No Access, Medium Access, High Access]
• Household wealth index	• A composite index of household possessions, assets and amenities [Low, Medium, High]
• Level of autonomy	• A composite variable showing woman’s participation in household decision making on health care, large household purchases, daily household purchases and mobility (visiting families and relatives) [Low Autonomy; High Autonomy]
***Community level***	
• Quality of health care index per province	• A composite index of the mean number of reproductive health services in the Province provided to women
• Provincial level of access to health care	• Proportion of Women in the Province experiencing at least one problem accessing health care
• Mean number of children per province	• Mean Number of Children ever born per woman in the Province
• Province level of women educational attainment	• Mean Number of Years of Female Education per women in the Province
• Province level of female with at least secondary education attainment	• Proportion of Women in the Province with at least secondary education
• Province level of approval of contraceptive use	• Proportion of women in the Province with partners approving of use of contraception
• Province level of contraceptive use	• Contraceptive Prevalence Rate in the Province
• Province level of ANC attendance	• Proportion of Women in the Province Accessing ANC at Least Once
• Province level of health facility use for delivery	• Proportion of Women in the Province who Delivered their Last Child in a Health Facility
Province level of women labour force participation	• Proportion of Women in the Province Participating in Labor Force

### Statistical analysis

The data from the 2010/11 ZDHS have information at different levels such as individual and household level data, and provincial data. The data therefore is hierarchical in nature. The data require techniques that offer a rigorous method for measuring the influence of community level contextual variables and unobserved contextual effects on use of modern contraception by adolescent women while providing a robust method for analyzing multilevel data [34,44,50]. The multilevel modeling technique facilitated the estimation of community level effects on adolescent women’s use of contraceptives and corrects the estimated standard errors to allow for clustering of observations within units [51] than the traditional regression method. This technique reduces chances of misestimating the significance of variables due to the traditional regression method.

The main outcome (dependent) variable (current use of modern contraceptives) of interest in this study is dichotomous. To account for the hierarchical structure of the data, a multilevel model technique was used as it allows for the identification of the random effect in the use of modern contraceptives by adolescent women. Since adolescent women are nested within provinces in Zimbabwe, it required the following combined hierarchical linear model formula:

$$\mathrm{Logit}\;\left[p_{\mathrm{ij}}\right]\;=\;\beta_{0}+\;\beta_{1}x_{\mathrm{ij}}+\;u_{j}$$

Where; p_ij_is the probability of the outcome for the i^th^ woman in the j^th^ province β is the vector of unknown parameters x_ij_ is the explanatory variable corresponding to the i^th^ woman in the j^th^ province u_j_ is the random effect at the province.

Adolescent women are at level 1 and they are nested within provinces which is level 2. Since this is a simple additive model of level 2 community level contextual variables, both levels (level 1 and level 2) were expected to have an equal intercept. Provincial level errors were thus included in the model and there was no random effect at the individual level. The use of provincial level estimates are appropriate since most of policy decisions influencing use of reproductive health services including use of modern contraception are taken at provincial level.

Binary logistic regression was applied for use of modern contraceptives using separate models. Model 1 is an empty model and model 2 included all individual-level variables to determine the level of variance explained by the model. Model 3 included the macro level contextual variables and Model 4 was the final model in which both the individual variables were put together with community (provincial) level contextual variables. To get to the final model, control variables that were significant in at least 1 of the models were first included to test for the fit of the final model. To improve the fit for the model, some of the control variables that were not significant but were considered biologically important in influencing utilization of modern contraceptives were also included in stages until a good fit was established. Random coefficients were tested to determine the coefficient model.

To perform the analysis, IBM SPSS Statistics software version 20 was used. Using the software, data was manipulated to come with univariate and bivariate analysis for the dependent variables. Multilevel modeling was fitted using the Generalized Linear Mixed Models (GLMM) (Hierarchical Generalized Linear Model). GLMM is an extension of generalized linear models such as logistic regression and its meant to include both the fixed and random effects, hence mixed models.

## Results

### Sample characteristics of adolescent women

Of the 457 adolescent women who had sex during the 12 months preceding the 2010/11 ZDHS survey, 77.2% were ever married (22.8% were never married) and 70.4% resided in rural areas. A majority of them were aged 18 to 19 years (64.2% with mean of 17.7 years), had high level of autonomy (59.5%), had a large age difference with their husbands or partners (68.7% with a mean of 7.5 years), had secondary or higher level of education (63%) and had given birth to one or more children (59.5%). The mean number of children for the sample was 0.66. The Traditional and Apostolic Sect religion constituted 43.7% whereas about a third (33.8%) belonged to either Protestants or Pentecostal. The Catholic, Muslim, non-affiliated and other Christian religions constituted 22.5% of adolescent women. About a quarter (25.4%) had high access to media and about two fifths (38.9%) had an average access to media. Slightly above a third of adolescent women (36.2%) had high household wealth. Those with medium and low household wealth constituted 21.9% and 41.9% of adolescent women respectively. Out of the 457 adolescent women, 39.4% indicated that they were current users of modern contraceptives (see Table 2).

**Table 2:** Percentage distribution of adolescent women who have had sex during 12 months preceding 2010/11 ZDHS by selected characteristics

**Characteristics**	**% (N = 457)**	**Characteristics**	**% (N = 457)**
**Age**		**Residence**	
15 to 17 years	**35.8**	Urban	**29.6**
18 to 19 years	**64.2**	Rural	**70.4**
*Mean age*	***17.7***	**Media access**	
**Marital status**		No access	**35.7**
Never married	**22.8**	Medium access	**38.9**
Ever married	**77.2**	High access	**25.4**
**Age difference with partner**		**Wealth index**	
Large	**68.7**	Low	**41.9**
Small/None	**31.3**	Medium	**21.9**
*Mean difference*	***7.5***	High	**36.2**
**Parity**		**Level of autonomy**	
None	**40.5**	Low autonomy	**40.5**
One or more pregnancies	**59.5**	High autonomy	**59.5**
*Mean no. of children*	***0.66***	**Contraceptive use**	
**Education**		Modern	**39.4**
None/Primary	**37.0**	Non-modern or none	**60.6**
Secondary or higher	**63.0**		
**Religious affiliation**		Total	**100.0**
Protestant/Pentecostal	**33.8**		
Traditional/Apostolic sect	**43.7**		
Catholic/Muslim/None/Other^ǂ^	**22.5**		

^ǂ^Other include any other Christian groups.

### Community (provincial) level characteristics of adolescent respondents

Data from the 2010/11 ZDHS reveal a significant variation in the community level characteristics of the respondents considered for this study. Table 3 shows means and ranges of the community level characteristics across Provinces or communities in Zimbabwe. For example, the quality of health care index which measured the average number of reproductive health care services considered rendered to women for each province ranged from 4.77 to 6.02.

**Table 3:** Means and ranges of community (Provincial) level characteristics across provinces in Zimbabwe

**Characteristic**	**Mean**	**Standard deviation**	**Range**
**Quality to health care**			
• Quality to health care index	**5.16**	**0.38**	**4.77 – 6.04**
**Access to health care**			
• % of women reporting at least one problem accessing health care	**62.0**	**10.17**	**42.9 – 74.5**
**Socioeconomic development**			
• Mean number of children ever born per woman	**2.1**	**0.32**	**1.43 – 2.52**
• Mean number of years of school for women	**8.9**	**0.81**	**7.70 – 10.28**
• % of women with at least secondary education	**67.8**	**11.03**	**52.2 – 87.2**
• % of women using contraceptives with partners approving of family planning	**96.8**	**1.33**	**91.5 – 98.6**
• Contraceptive prevalence rate	**57.4**	**3.92**	**45.2 – 61.6**
• % of women accessing ANC at least once	**89.7**	**3.00**	**86.7 – 95.9**
• % of women who delivered their last child in a health facility	**64.6**	**11.30**	**50.3 – 88.8**
• % of women participating in labour force	**43.9**	**12.45**	**19.2 – 60.6**

Source: 2010/11 Zimbabwe demographic health survey.

Access to health care was measured using wide range of areas such as ability to go for treatment, affordability, distance to health facility and mobility (transportation) by women across provinces. The proportion of women who report experiencing at least one of the problems in the various areas considered in each province varied from 42.9% to 74.5%. With regard to socioeconomic development indicators, the mean number of children ever born per women for provinces ranged from 1.43 to 2.52 whereas the mean number of years of school for women ranged from 7.70 to 10.28. The proportion of women with at least secondary education ranged from 52.2 to 87.2. The range for the proportion of women reporting husbands/partners approving of family planning and those accessing ANC at least once was 91.5%-98.6% and 86.7%-95.9% correspondingly. Provincial contraceptive prevalence rate varied from 45.2% to 61.6%. The proportion of women who delivered their last child in a health facility varied from 50.3% to 88.8%. Participation of women in labour force ranged from 19.2% to 60.6%. As indicated in Table 3, there were significant variations between provinces on these community level variables.

### Bivariate analysis

Contraceptive use among adolescent women increased with age. Adolescent women aged 18 to 19 year old were almost 1.5 times more likely to use contraceptives compared adolescent aged 15 to 17 years (44% vs. 31.1%) with mean age of modern contraceptive users at 18.0 years. Close to half of the ever married (44.6%) were current contraceptive users as compared to 21.1% of the never married. The proportion of adolescent women with one or more children ever born currently using contraceptives was eight (8) times higher than that of adolescent women with no children ever born (61% vs. 7.6%).

The proportion of adolescent women with a large age difference with their husbands/partners who are current contraceptive users was higher with 42.4% as compared to 34.7% among adolescent women with a small or no age difference. Modern contraceptive use among adolescent women residing in rural areas was higher than for those residing in urban areas (41.9% vs. 33.3%). The level of education, religion, level of media access, level of autonomy and household wealth index were not statistically associated with modern contraceptive use of adolescent women (see Table 4). A test for collinearity indicated that none of the variables were significantly correlated.

**Table 4:** Means and percentage of adolescent women who had sex during the 12 months preceding the 2010/11 ZDHS using modern contraceptives by selected characteristics

**Characteristics**	**% (N = 457)**	**Characteristics**	**% (N = 457)**
Age**		**Religious affiliation**	
15 to 17 years	**31.1**	Protestant/Pentecostal	**33.8**
18 to 19 years	**44.0**	Traditional/Apostolic sect	**42.5**
*Mean age*	***18.0***	Catholic/Muslim/None/Other^ǂ^	**38.8**
Marital status***		Residence*	
Never married	**21.2**	Urban	**33.3**
Ever married	**44.6**	Rural	**41.9**
Age difference with partner*		**Media access**	
Large	**42.4**	No access	**35.6**
Small/none	**34.7**	Medium access	**41.6**
*Mean age difference*	***7.5***	High access	**41.4**
Parity***		**Wealth index**	
None	**7.6**	Low	**40.3**
One or more pregnancies	**61.0**	Medium	**42.0**
*Mean no. of children*	***1.02***	High	**37.0**
**Education**		**Level of autonomy**	
None/Primary	**43.8**	Low autonomy	**38.9**
Secondary or higher	**36.8**	High autonomy	**40.2**

*= p < 0.05; **= p < 0.01; ***= p < 001.

^ǂ^Other religions include any other christian religion.

### Multilevel analysis (multivariate)

The first model (model 1) which is an empty model, investigated the intercept and the random variance component of the intercept. Results show a variance coefficient of 0.715 which was significant (see Table 5). This means that a normally distributed random intercept of adolescent women random contraceptive use has a variance of 0.715. The model had no micro level individual and community level contextual variable indicating that the differences in adolescent contraceptive use between provinces (community) may be attributable to either unobserved micro level variables or other unobserved community level contextual variables.

**Table 5:** Multilevel odds ratios assessing effects of individual and community characteristics on contraceptives use among adolescent women who had sex during the 12 months preceding the survey in Zimbabwe, 2010/11 ZDHS

**Variables**	**Model 1**		**Model 2**		**Model 3**		**Model 4**	
	**Coefficients β (SE)**	**Odds ratio**	**Coefficients β (SE)**	**Odds ratio**	**Coefficients β (SE)**	**Odds ratio**	**Coefficients β (SE)**	**Odds ratio**
**Intercept**	-2.292 (0.736)***		-1.690 (0.659)		-1.396 (0.156)		-2.122 (0.657)	
**Random intercept variance**	0.715 (0.272*)		0.328 (0.298)		0.482 (0.566)		0.302 (0.846)	
**PCV (%)**	Reference		54.1		32.6		57.8	
**INDIVIDUAL LEVEL**								
**Age**								
15 to 17 years	NI	NA	1					
18 to 19 years	NI	NA	0.288 (0.266)	1.334			0.173 (0.292)	1.189
**Marital status**								
Never married	NI	NA	1				1	
Ever married	NI	NA	0.852 (0.254)	2.344***			0.908 (0.244)	2.479***
**Parity**					NI	NA		
None	NI	NA	1					
One or more pregnancies	NI	NA	2.809 (0.250)	16.593***			2.610 (0.272)	13.6***
**Age difference with spouse**					NI	NA		
Large difference	NI	NA	1				1	
Small difference/None	NI	NA	-0.253 (0.253)	0.776			-0.106 (0.280)	0.899
**Education**								
None/Primary (RC)	NI	NA	1				1	
Secondary or higher	NI	NA	-.005 (0.263)	0.995	NI	NA	0.062 (0.292)	1.064
**Religion**								
Protestant/Pentecostal	NI	NA	1				1	
Traditional/Apostolic sect	NI	NA	-0.112 (0.279)	0.829	NI	NA	-0.416 (0.306)	1.516
Catholic/Muslim/None/Others^ǂ^	NI	NA	-0.030 (0.341)	0.970	NI	NA	-0.023 (0.373)	0.977
**Residence**								
Urban	NI	NA	1				1	
Rural	NI	NA	-0.274 (0.323)	0.761	NI	NA	-0.041 (0.565)	0.960
**Media access**								
No access	NI	NA	1				1	
Average access	NI	NA	0.459 (0.221)	1.582*	NI	NA	0.598 (0.232)	1.818*
High access	NI	NA	0.550 (0.254)	1.733**	NI	NA	0.758 (0.328)	2.134**
**Household wealth index**								
Low	NI	NA	1				1	
Medium	NI	NA	-0.143 (0.317)	0.867	NI	NA	-0.110 (0.345)	0.896
High	NI	NA	-0.393 (0.365)	0.675	NI	NA	-0.179 (0.406)	0.836
**Level of autonomy**								
Low autonomy	NI	NA	1					
High autonomy	NI	NA	-0.077 (0.235)	0.926	NI	NA	-0.046 (0.259)	0.955
**COMMUNITY LEVEL**								
**Quality of health care**								
• Quality of health care index	NI	NA	NI	NA	0.081 (0.606)	1.084	-0.178 (0.845)	0.837
**Access to health care**								
• % of women reporting at least one serious problem accessing health care	NI	NA	NI	NA	0.391 (0.131)	1.478*	0.686 (0.212)	1.986*
**Socioeconomic development**								
• Mean number of children ever born per woman	NI	NA	NI	NA	-3.008 (1.062)	0.049**	-2.647 (1.028)	0.071***
• Mean number of years of school for women	NI	NA	NI	NA	-0.706 (0.151)	0.494**	-0.986 (0.305)	0.373**
• % of women with at least secondary education	NI	NA	NI	NA	-0.684 (0.192)	0.504**	-0.787 (0.234)	0.455**
• % of women with husbands/Partners approving of family planning	NI	NA	NI	NA	0.156 (0.282)	1.169	0.436 (0.972)	1.546
• Contraceptive prevalence rate	NI	NA	NI	NA	0.387 (0.141)	1.473*	0.252 (0.338)	1.287
• % of women accessing ANC at least once	NI	NA	NI	NA	-0.411 (0.269)	0.663	-0.363 (0.674)	0.699
• % of women who delivered their last child in a health facility	NI	NA	NI	NA	-0.054 (0.076)	0.947	-0.067 (0.168)	0.935
• % of women participating in labour force	NI	NA	NI	NA	0.016 (0.022)	1.013	0.045 (0.054)	1.046

*= p < 0.05; **= p < 0.01; ***= p < 001^ǂ^Others includes other christian religions not mentioned.

Model 2 included the micro level individual and household variables only. This model correctly predicted 77.5% of the cases. Having one or more children ever born by adolescent women increased the odds of using modern contraceptive methods (Odds Ratio, 16.6). The ever married were more than twice as likely to be current contraceptive users as the never married (Odds ratio, 2.3). Odds of current contraceptive use increased with an increase in the level of media access. Adolescent women with medium and high access to media were 1.6 and 1.7 times more likely to adopt modern contraceptive use than those with no access to media. Surprisingly, level of education, level of autonomy and household wealth index were not associated with adolescent women contraceptive use. The model has a variance of the random intercept of 0.328 which is not significant (see Table 5). This means that individual level variables in the model explained some of the variation in adolescent contraceptive use between provinces (clusters). Some of the between cluster variation may also be explained by some unobserved individual characteristics or community level characteristics.

Model 3 included the provincial (community) level variables but did not include the micro level individual and household level variables. The community (province) quality of health care index was not associated with adolescent women’s use of modern contraceptives. The odds of adolescent women using modern contraceptives increased with the community (province) proportion of women reporting at least one serious problems accessing health care (Odds Ratio, 1.5). The model showed that residing in a community with a higher mean number of children ever born per woman was associated with lower odds of modern contraceptive use (Odds Ratio, 0.049).

Results in Table 5 also show some peculiar relationships which may indicate some element of compositional effects of education on contraceptive use among adolescent women. Residing in a community (province) with a higher mean number of years of school for women was associated with lower odds of modern contraceptive use among adolescent women (Odds Ratio, 0.5). The odds of using a modern contraceptive method among adolescent women also lessened with residing in a community with higher proportion of women with at least secondary school education (Odds Ratio, 0.5). The results may also indicate that individual adolescents may not necessarily be influenced to use contraceptives by the effects of high level of educational attainment among women in the community. The higher the community (provincial) contraceptive prevalence rate, the higher the odds of modern contraceptive use by adolescent women (Odds Ratio, 1.5). The variance of the random intercept for this model had been increased to 0.482 from that of model 3 and it was not significant. This indicates that model 2 is a better explanatory model than model 3 but unexplained variation between provinces still remains.

Model 4 which is the final model included both the micro level individual and household variables as well as community (provincial) level variables. The association of individual and household level variables was almost similar to that of model 2 whereas the effect of one of the community (provincial) level variables was eliminated. At individual level, contraceptive use increased with having average and high access to media (OR, 1.8; 2.1 respectively). Contraceptive prevalence rate at community level was no longer associated with adolescent women modern contraceptive use after controlling for the effects of individual level variables. The effect of quality of reproductive health care on adolescent women modern contraceptive use remains insignificant after controlling for individual level variables.

Among community (provincial) level characteristics, only four of the five that were significant on model 3 remained significant. The odds of modern contraceptive use by adolescent women were higher in provinces that had a higher proportion of women reporting at least one major problem accessing health care (Odds Ratio, 2.0). Adolescent women living in communities with a higher mean number of children ever born per women were less likely to be modern contraceptive users (Odds ratio, 0.07). Residing in a community with a higher mean number of years of school for women was associated with lower odds of using a modern contraceptive method by adolescent women (Odds Ratio, 0.4). The odds of using a modern contraceptive method were also lessened by residing in a community with a higher proportion of women with at least secondary school education (0.5).

Putting community level variables in the model and controlling for individual and household level variables reduced the variance of the random intercept to 0.302 (Table 5). The random intercept is not significant indicating homogeneity across clusters even after both micro level individual and community level contextual variables are considered. In conclusion, both individual level and community level characteristics are important predictors of adolescent contraceptive use. However, there still remain unobserved individual and community (provincial) level characteristics that are associated with adolescent use of modern contraceptives.

## Discussions

This study examined the influence of individual, household and community characteristics in adolescent women contraceptive use in Zimbabwe. Individual characteristics which were influential on adolescent women use of modern contraceptives included parity, marital status and access to media and this is similar to findings from other studies [16,25]. Community characteristics that had independent effect on adolescent contraceptive use included provincial mean number of children ever born per woman, provincial mean number of years of school for women, provincial proportion of women with at least secondary education and provincial access to health care.

Parity seemed to have the most effect on adolescent contraceptive use and this is followed by marital status. Adolescent women with at least one least one child and those that were ever married were more likely to be users of contraceptives. Findings indicate that about three quarter of sexually active adolescent women in Zimbabwe were married indicating a high probability of marriage for any adolescent women on sexual debut. Adolescent women may be expected to prove their fertility potential once they become sexually active regardless of the reproductive health outcomes. The urge to have a first child is coming out to be the most effective factor influencing use of modern contraceptives. Adolescent women with zero parity were eight times less likely to use modern contraceptives as compared to those with one or more children ever born. The results do establish that once an adolescent has achieved motherhood, then the likelihood of modern contraceptive use increases. The need to delay sexual debut of adolescent women through involvement of communities becomes an important policy priority especially in societies that encourage early age marriages. The influence of religion may also be linked to this but it has been found not to have any significant influence on contraceptive use in this study. Religious affiliation has been recognized as influential to fertility behaviour in other studies [51,52]. The lack of association in this study may be attributed to the categorization of variables which were influence by the size of other religions such as the Catholics.

Findings also reveal that access to media does influence adolescent contraceptive use. Media access was measured using a composite variable created from exposure to newspaper, radio and television. The influence of access to media on contraceptive use increased with the level access to media. Other exposures to family planning messages through other means were not considered for this study and hence this might bias the effect of media access on contraceptive use. This finding however is in line with other studies that associated exposure to family planning messages with contraceptive use [13,17,21,25].

Other individual socio-economic variables such as residence, household wealth index and level of autonomy did not have any influence on adolescent contraceptive use as would have been expected. This study does demonstrate that adolescent woman’s socio-economic circumstances, such as education, wealth, and level of autonomy may not independently be accurate predictors of contraceptive use as also shown in other studies [11,12,43]. The influence of education on contraceptive use may be compromised by considerable diffusion effects in progress which may operate at levels beyond the adolescent women’s circumstances. A similar diffusion effect on contraceptive use by uneducated women was found in India where mass media exposure emerged as an important diffusion channel [43]. A similar explanation can be said with regard to the lack of association between contraceptive use and household wealth and residence contrary to existing literature [15]. Clearly, the need to look beyond just the individual socioeconomic women’s circumstances is justified here.

The women’s level of autonomy on the other hand was measured using observable indicators reflecting various aspects of a women’s position in the household. The lack of association at both bivariate and multivariate analysis is contrary to previous studies [12,18,19]. This finding may reflect the community norms that sometimes pressurize adolescents to perform their expected cultural reproductive duties of bearing children once they become sexually active. This is an important factor and is reflected by a significant association between parity and contraceptive use even after controlling for community level variables. The need to have a child once an adolescent becomes sexually active is the driving force behind non-use of contraceptive among adolescents and this is in line with findings in other studies [20]. Among the adolescent women who were in a sexual union, none of those with no children ever born were current contraceptive users whereas about 60% of those with one or more children ever born were contraceptive users (*not shown in the results)*. This may indicate that once an adolescent has proven their fertility, use of contraceptive may be initiated. This may also explain why being ever married increased odds of contraceptive use.

Measuring autonomy for this study on the other hand did not consider including decision making on use of contraceptives and family planning as one of the aspects of a woman’s position in the household. In Zimbabwe, the use of contraceptives and decision on family planning remains the subject to a man’s willingness [53]. However, community level interventions which are human rights based and aimed at preventing unintended pregnancies should be considered as they have been found to be effective in some settings [54]. Such interventions should involve use of targeted, enabling and catalytic approaches in programming for social change in a range of sectors.At community level, adolescent women residing in communities with higher number of children per woman were less likely to be using modern contraceptives. This may be an indication of pro-natalist community norms that may still be entrenched in some societies in Zimbabwe. Adolescent women residing in communities with high level fertility are more likely to be influenced to have a desire to prove their fertility.

Education levels at community level play a peculiar role in influencing adolescent reproductive behaviour in this study. Whilst education has been known to positively influence modern contraceptive use, adolescent women residing in communities with higher number of years of school per women and in communities with a higher proportion of women with secondary education were less likely to be current contraceptive users. These findings differ with those from other studies that linked increased level of education with contraceptive use [11,12]. The level of education does not seem to mediate the pro-natalist norms ingrained in Zimbabwe among women and this is passed to adolescent women even if they are educated. Once adolescent women get into a sexual union, it is thus expected that they show their fertility potential [55]. The use of contraceptives becomes a necessity after bearing a child, a proof that one is potentially fertile. This anomaly calls for the need to further research on the influence of other community level influences which may mediate the relationship of contraceptive use with education levels. This may be either political or cultural factors or the presence of specific diffusion effects within communities.

The positive influence of health service characteristics on contraceptive use has been revealed in other studies [26-30]. This study however used nine reproductive health service characteristics to measure quality of health care. Unlike in other studies, quality in health care at community level did not influence use of contraceptives by adolescent women. However, adolescent women residing in a community with a higher proportion of women reporting at least one problem accessing health care more likely to be modern contraceptive users. This is corroborated by other studies which associated access to reproductive health with use of contraceptives [28]. It also demonstrates that even if adolescent staying in an environment where women experience hardships in terms of accessing health care, the use of modern contraceptives may still be influenced by other factors.

In conclusion, both the individual and community level variables included in the models do fully explain the variation in adolescent contraceptive use across provinces in Zimbabwe. At individual level, having at least one child, being ever married and having access to media increased odds of using contraceptives among adolescent women. At community level, having a higher mean number of children ever born per women, having a higher mean number of years of years of school for women and having a higher proportion of women with at least secondary education decreased odds of contraceptive use among adolescent women. However, having a higher proportion of women reporting at least one serious problem accessing health increased odds of contraceptive use. Other community level variables such as socio-cultural and political factors need to be considered when studying adolescent contraceptive use.

It should be noted that individual level variables considered do explain some of the variations much better than community level variables considered. The random intercept variance for community level variables model considered was higher than that of individual and household level variables and not significant. However, the final model which combined both individual and household level variable with the community level variables had a better fit than model 2 and model 3. This is an indication that both individual and community level variables should be considered when studying determinants of contraceptive use among adolescent women. In addition, further studies using qualitative approaches should be carried out to explain some of the associations or lack of associations revealed in quantitative multilevel models. This study informs policy decisions and suggests that interventions aimed at reducing unintended pregnancies among adolescent women should not only look at individual level characteristics but also community level social context.

### Limitations

The main goal of the study was to examine the influence of community level variables on adolescent contraceptive use and community level variables considered were limited to the quality of reproductive health care, access to health care and socioeconomic development indicators. Other important community characteristics such as political and cultural factors were not covered. This may have possibly explained some of the variations across provinces that were still not explained by the study.

Data used to measure quality of health care was only limited to 9 reproductive health services and other aspects of quality such as patients’ perspectives and infrastructural factors were not covered. Access to health care at community level was measured using data from reports of personal experience or perspectives on areas of getting permission to go for treatment, getting money for treatment, distance to a health facility, and having to take transportation. Other technical areas such as physical access and topography were not considered. Similarly, the aspect of access to media was only limited to newspaper, radio and television whereas areas such as exposure to family planning messages were not considered and hence this might bias the effect of media access.

Another limitation to the study is the use of religion instead of pro-natalist cultural values related to some religions which may have been important as characteristics of such communities in which marriages take place with young girls. Some religions such as African Traditional and Apostolic Sect encourage polygamy and usually deny use of formal health services to their members.

This is a cross-sectional survey and thus the temporal order of contraceptive use could not be determined. Associations established by the study do not therefore show any causality relationship. However, a review of cross-sectional data is useful for making hypotheses which would need further research to corroborate the findings from this study using methods designed to clarify some of the relationships revealed by the study. One other limitation is that the use of Provinces as a unit of analysis for multilevel approach may be too large and thus may provide a measurement problem. It should be noted that current use of contraceptives may be influenced by more proximal factors that affect the day to day life and activities of the adolescent women [56].

## Competing interests

Both authors declare that they have no competing interests.

## Authors’ contributions

Both EN and CO conceived the idea and planned the study. EN acquired secondary data from Measure DHS and did the analysis, and interpretation of data. Both EN and CO worked together in drafting and revising the manuscript critically for important intellectual content. They both read and approved the final manuscript.

## Authors’ information

EN is a lecturer in the Department of Population Studies of the University of Botswana, Gaborone, Botswana. He is currently pursuing PhD studies in the Demography and Population Studies Programme of the University of the Witwatersrand in Johannesburg, South Africa. CO is a Professor and head of the Demography and Population Studies Programme of the University of the Witwatersrand.
